# Orphan G-Protein Coupled Receptor 22 (Gpr22) Regulates Cilia Length and Structure in the Zebrafish Kupffer’s Vesicle

**DOI:** 10.1371/journal.pone.0110484

**Published:** 2014-10-21

**Authors:** Daphne Verleyen, Frank P. Luyten, Przemko Tylzanowski

**Affiliations:** 1 Department of Development and Regeneration, Laboratory for Developmental and Stem Cell Biology, Skeletal Biology and Engineering Research Centre, University of Leuven, Leuven, Belgium; 2 Department of Biochemistry and Molecular Biology, Medical University, Lublin, Poland; National Eye Institute, United States of America

## Abstract

GPR22 is an orphan G protein-coupled receptor (GPCR). Since the ligand of the receptor is currently unknown, its biological function has not been investigated in depth. Many GPCRs and their intracellular effectors are targeted to cilia. Cilia are highly conserved eukaryotic microtubule-based organelles that protrude from the membrane of most mammalian cells. They are involved in a large variety of physiological processes and diseases. However, the details of the downstream pathways and mechanisms that maintain cilia length and structure are poorly understood. We show that morpholino knock down or overexpression of *gpr22* led to defective left-right (LR) axis formation in the zebrafish embryo. Specifically, defective LR patterning included randomization of the left-specific lateral plate mesodermal genes (LPM) (*lefty1*, *lefty2*, *southpaw* and *pitx2a*), resulting in randomized cardiac looping. Furthermore, *gpr22* inactivation in the Kupffer’s vesicle (KV) alone was still able to generate the phenotype, indicating that Gpr22 mainly regulates LR asymmetry through the KV. Analysis of the KV cilia by immunofluorescence and transmission electron microscopy (TEM), revealed that *gpr22* knock down or overexpression resulted in changes of cilia length and structure. Further, we found that Gpr22 does not act upstream of the two cilia master regulators, Foxj1a and Rfx2. To conclude, our study characterized a novel player in the field of ciliogenesis.

## Introduction

G-protein-coupled receptors (GPCRs) represent the largest protein superfamily in the human genome, including at least 800 receptors with a common seven-pass transmembrane domain organization. A large variety of ligands can bind and activate these receptors, such as light-sensitive compounds, pheromones, hormones, neurotransmitters, ions, peptides, proteins and many more. The endogenous ligands for more than 140 GPCRs remain however unidentified making them ‘orphan’ receptors [1], [2]. The current model of the receptor activation proposes that ligand binding induces a conformational change, changing the interaction with intracellular heterotrimeric G-proteins, consisting of a Gα and a Gβγ subunit. Subsequently, the receptor catalyzes the exchange of GDP to GTP in the Gα subunit. Next, the activated G protein subunits (α-GTP and βγ) regulate many downstream effectors, like adenylate cyclases, RhoGTPases and phospholipases [1]. In addition, GPCRs bind to β-arrestins and GPCR kinases (GRKs), which can activate G-protein independent signaling pathways, such as JNK, RhoA, MAPK or ERK [3]. Moreover, GRKs and β-arrestins are involved in GPCR desensitization by preventing G-protein coupling and stimulating endocytosis of the receptor [4], [5].

GPR22 is an orphan G-protein-coupled receptor, belonging to the class A rhodopsin-like GPCRs. The receptor was discovered together with three other human GPCR genes, using a customized search of a database of expressed sequence tags. RNA expression analysis revealed the presence of *GPR22* in several human brain regions, such as the cerebellum, cerebral cortex and amygdale [6]. Furthermore, immunohistochemistry showed that GPR22 was present in cardiac myocytes and coronary arteries. *Gpr22* null mice did not show any detectable phenotype, but had a significantly increased risk to functional cardiac decompensation following aortic banding. Overexpression of *GPR22* in HEK-293 cells revealed that the receptor coupled to Gαi/Gαo, indicating that activation of the receptor would lead to the inhibition of adenylyl cyclase, a decreased production of cAMP and a decreased PKA activity [7]. Genome-wide association scan (GWAS) identified an osteoarthritis (OA) susceptibility locus on chromosome 7q22 that contains 6 genes, including *GPR22* [8],[9]. In mice, GPR22 was expressed in the chondrocytes of the articular cartilage of knee joints after the induction of arthritis by papain or methylated bovine serum albumin and in the osteophytes of mice with instability-induced OA [8]. Nonetheless, the biological function and regulation of *GPR22* remains poorly investigated and its role in the disease process is still debated [8]–[10].

GPCRs regulate many processes such as neuronal development, cardiac development, left-right (LR) patterning, cell division, inflammation and sensation (vision, taste, smell) [1], [11]. At the subcellular level, many GPCRs and their intracellular effectors are targeted to cilia [12]–[15]. Cilia are highly conserved eukaryotic organelles that protrude from the cell’s plasma membrane. They can be divided into 2 types, the primary immotile cilia and the motile cilia. Primary cilia are present on nearly every mammalian cell and are involved in the signal transduction of many pathways, involving HH, Wnt or GPCR pathways. Primary cilia consist of nine parallel outer microtubule doublets (‘9+0’). Motile cilia mostly have an additional central pair of microtubuli (‘9+2’), as well as radial spokes and dynein arms [16]–[18]. However, the zebrafish Kupffer’s vesicle also contains motile cilia that lack the central pair [16]. The main function of the motile cilium is to create a fluid flow, for example to move the fertilized egg into the oviduct, to circulate the cerebro-spinal fluid in the brain ventricles or to induce LR asymmetry in the embryo [17]–[19].

The zebrafish embryo is a commonly used *in vivo* model to study ciliogenesis. They develop *ex utero*, are transparent, easy to manipulate and develop many ciliated organs during early embryogenesis. Importantly, there are many well-characterized zebrafish ciliary mutants available. Typical characteristics of these ciliary mutants are a downwards curvature of the tail and LR patterning defects [20]–[22]. Zebrafish LR asymmetry is induced by a ciliated transient organ called the Kupffer’s vesicle (KV), which is similar to the mouse embryonic node. The KV is formed at early somitogenesis by the dorsal forerunner cells (DFCs) that form a ciliated epithelium around a fluid-filled lumen. The cilia inside the KV are motile and create a directional fluid flow directing asymmetric gene expression (for example *southpaw, pitx2a, lefty 1/2*) in the left lateral plate mesoderm (LPM). These genes induce the LR asymmetry of the developing organs, like the heart and the liver [23], [24]. Consequently, cilia defects often result in defective LR axis formation.

At the transcriptional level, two transcription factors act as master regulators of ciliogenesis, Foxj1 (forkhead box J1) and Rfx2 (regulatory factor X). They control the expression of genes encoding components necessary for cilia assembly or function, like dyneins, kinesins and tektins [25]. However, the details of the downstream pathways and mechanisms that maintain cilia length and structure are poorly investigated. The importance of identifying these regulators is underscored by many physiological processes and diseases, known as ciliopathies, in which cilia are involved.

In this study, we show that deregulation of *gpr22* in the zebrafish embryo led to defective left-right (LR) patterning, caused by defects in KV cilia length and structure. Furthermore, our study indicates that Gpr22 does not act upstream of Foxj1a and Rfx2. Thus, Gpr22 is a novel player in the field of ciliogenesis, acting independently or downstream of the cilia master regulators.

## Results

### Deregulation of *gpr22* results in a curvature of the tail and heart edema

**Figure 1:**
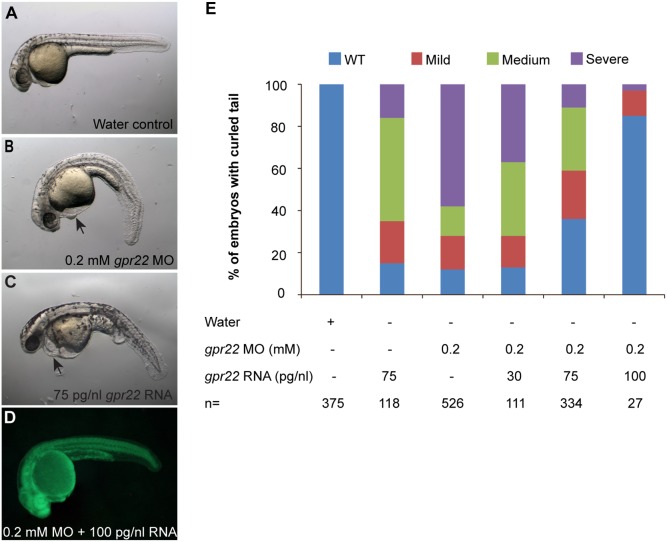
The downwards bending of the tail in *gpr22* morphants is rescued by the co-injection of *gpr22* mRNA. (**A–C**) Lateral views with anterior to the left of zebrafish embryos at 24 hpf injected at one cell stage with (**A**) water, (**B**) 0.2 mM fluorescein tagged *gpr22* MO, (**C**) 75 pg/nl *gpr22* mRNA or (**D**) 0.2 mM fluorescein tagged *gpr22* MO and 100 pg/nl *gpr22* mRNA. (**B, C**) Deregulation of *gpr22* results in a bending of the tail, a reduction in length of the anterior-posterior axis and heart edema *(arrow)*. (**E**) Quantification of the tail phenotype. (**D, E**) The curly tail of embryos injected with fluorescein tagged *gpr22* MO is rescued by the co-injection of *gpr22* mRNA in a dose-dependent manner. MO = morpholino, hpf = hours post-fertilization, n = number of analyzed embryos, WT = wild type.

We cloned the zebrafish orthologue of human *GPR22* (NCBI reference sequence zebrafish: NM_001044765.1; human: NM_005295.2). The amino acid sequence of zebrafish Gpr22 (NP_001038230.1) showed 78% overall identity to the human protein (NP_005286.2). The most conserved regions were found in the transmembrane domains (TM) (88% identity), the intracellular loops (IL) (84% identity) and the C-terminal tail (86% identity). Further comparison revealed conserved motifs reported to play a role in GPCR activation and signaling, like the DRY motif between TM3 and IL2 and the PxxY motif in the TM7 domain [26], [27]. To investigate the biological function of GPR22, we performed gain of function (GOF) and loss of function (LOF) studies in the zebrafish embryo. The LOF studies were carried out by injecting morpholino (MO) into one cell stage zebrafish embryos. As a control for potential off-target effects of the MO, we used two translation blocking MOs (a fluorescein tagged MO1 and a non-tagged MO2), each targeting different sequences at the 5′UTR of *gpr22*. Zebrafish *gpr22* was first annotated as a single exon coding gene in the ZFIN, NCBI and Ensembl databases. Currently, the information provided by the databases about the splicing events of zebrafish *gpr22* is inconsistent. Therefore, we decided not to use splicing blocking MOs. Injection with either of the MOs resulted in the same dose-dependent phenotypes, but MO1 appeared to be more efficient, inducing a phenotype at 0.2 mM instead of 0.4 mM (**Fig. S1**). Consequently, we used 0.2 mM of MO1 (further referred to as MO) for all subsequent experiments, unless otherwise specified. Since MO1 caused p53-mediated apoptosis in the head, a well-known off-target effect of MOs, all silencing experiments were performed in the presence of 0.1 mM of p53 MO (**Fig. S1B, C**) [28]. At 24 hpf, a knock down of *gpr22* resulted in a downwards curvature of the tail, a reduction in length of the anterior/posterior (A/P) axis and heart edema. To ensure that the MO-induced tail phenotype was caused by a specific reduction of Gpr22 levels and not by off-target effects of the MO, we performed a rescue experiment by co-injecting fluorescein-tagged MO and capped, polyadenylated *gpr22* mRNA into one cell stage zebrafish embryos. The green fluorescence of the MO allowed us to distinguish the rescued WT morphants from the WT embryos that were not efficiently injected with MO (**Fig. 1D**). Co-injection of *gpr22* mRNA rescued the curly tail phenotype of the morphants in a dose-dependent manner (**Fig. 1**). The GOF experiments were carried out by the injection of the same *gpr22* mRNA into one cell stage zebrafish embryos. Injection of 75 pg/nl of *gpr22* mRNA induced a curly tail phenotype, a reduction in length of the anterior/posterior (A/P) and heart edema at 24 hpf. The phenotype was dose-dependent (**Fig. 1** **; Fig. S2**).

### Deregulation of *gpr22* results in defective LR patterning

**Figure 2:**
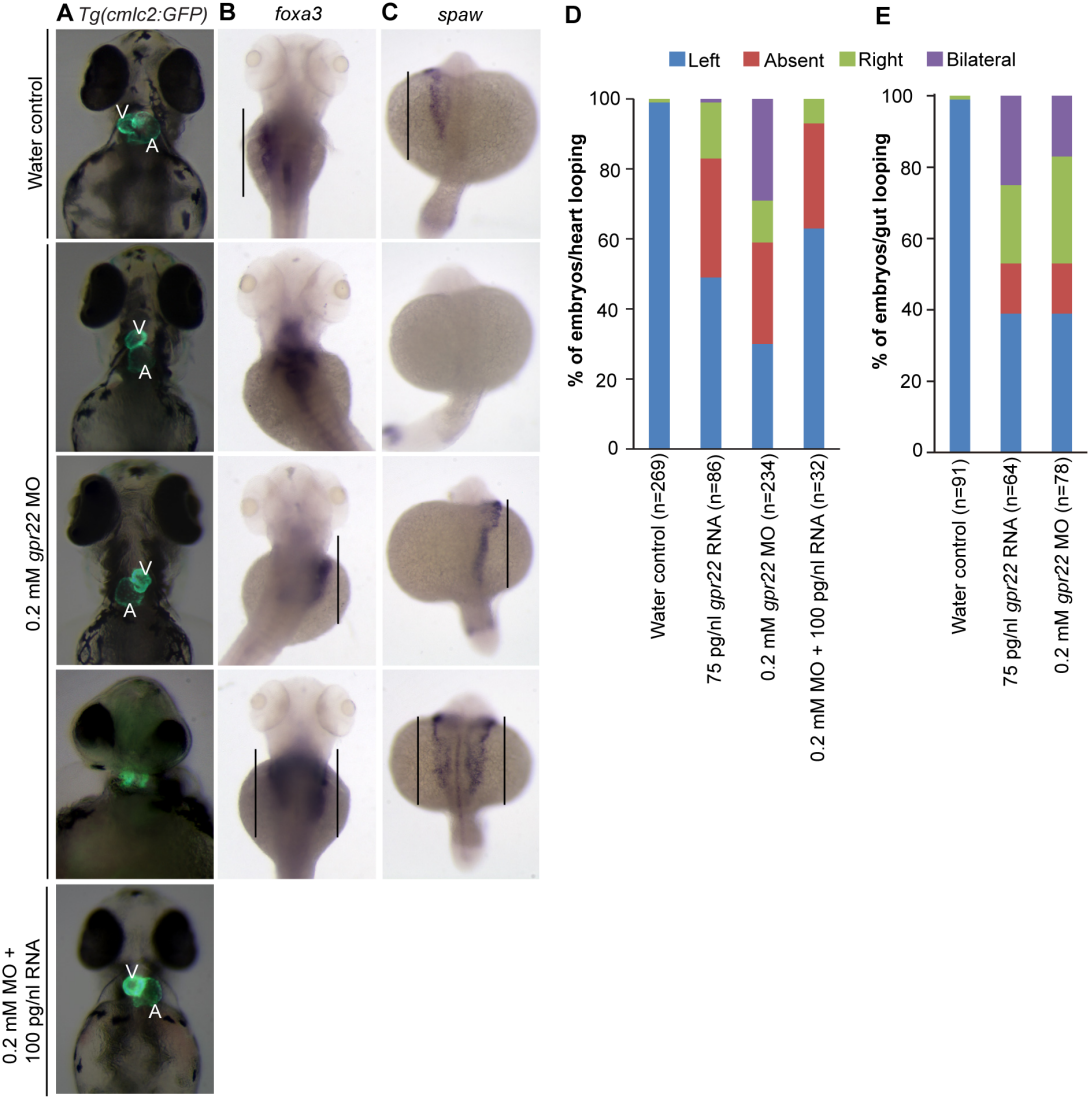
*gpr22* deregulation results in defective LR patterning. (**A**) Ventral or (**B, C**) dorsal views with anterior to the top of embryos at (**A, B**) 4 dpf or (**C**) 20 somite stage. (**A**) Transgenic embryos expressing GFP under control of the *cmlc2* promoter. In contrast to water controls, *gpr22* morphants show (from top to bottom) normal, absent, reversed or bicardial cardiac looping. Co-injection of 0.2 mM *gpr22* MO and 100 pg/nl *gpr22* RNA partially rescues the randomized cardiac looping (last panel). (**B**) WISH for *foxa3*. The liver of water control embryos develops at the left side of the embryo. In contrast, the position of the liver in embryos injected with 0.2 mM *gpr22* MO is randomized *(straight line)*. (**C**) WISH for *southpaw (spaw)*. *gpr22* morphants show randomized expression of the early left-specific LPM marker *southpaw (straight line)*. (**D**) Quantifications of the heart or (**E**) liver/gut phenotype. MO = morpholino, V = ventricle, A = atrium, dpf = days post-fertilization, n = number of analyzed embryos, GFP = green fluorescence protein, *cmlc2* = *cardiac myosin light chain type 2*, LR = left-right, WISH = whole mount in situ hybridization, LPM = lateral plate mesoderm.

The downwards curvature of the tail and the heart edema are often associated with defects in ciliogenesis [22]. One of the most common consequences of defective ciliogenesis during development is a defective left-right (LR) asymmetry of the developing organs, e.g. the heart, liver or gut [22], [24]. To investigate the LR asymmetry of the heart, we injected *gpr22* MO in transgenic zebrafish expressing green fluorescence protein (GFP) under the control of the cardiac myosin light chain 2 (*cmlc2*) promoter [29]. The LR asymmetry was defective in *gpr22* morphants, which showed a normal, reversed, absent or bicardial cardiac looping at 4 dpf (**Fig. 2A, D**). Importantly, the heart phenotype could be rescued by the co-injection of *gpr22* RNA (**Fig. 2A, D**). The position of the liver/gut was also randomized as visualized by whole mount in situ hybridization (WISH) for the liver/gut marker *foxA3* (**Fig. 2B, E**). Finally, a WISH for the left-specific LPM markers *southpaw* (the zebrafish *nodal*), *lefty1* and *pitx2a* at the 20 somite stage confirmed the randomized LR asymmetry in *gpr22* morphants (**Fig. 2C** **; Fig. S3;** **Table 1**). Embryos with an overexpression of *gpr22* also showed randomization of the left-specific LPM markers and consequently, a randomized cardiac looping and localization of the liver (**Fig. 2D, E** **; Fig. S4;** **Table 1**). These results suggest a possible function for Gpr22 in KV ciliogenesis.

**Table 1:** Randomized expression of the left-specific LPM markers in zebrafish with deregulated *gpr22* expression.

	*spaw*					*lefty1*				
	n	L (%)	R (%)	A (%)	B (%)	n	L (%)	R (%)	A (%)	B (%)
Water control	64	88	0	6	6	77	67	0	33	0
75 pg/nl *gpr22* RNA	24	42	4	50	4	83	50	7	40	3
0.2 mM *gpr22* MO	41	39	7	24	30	94	14	5	77	4
	***pitx2a***									
	**n**	**L (%)**	**R (%)**	**A (%)**	**B (%)**					
Water control	95	83	0	17	0					
75 pg/nl *gpr22* RNA	82	47	6	35	12					
0.2 mM *gpr22* MO	101	34	16	34	16					

MO = morpholino, n = number of analyzed embryos, L = left, R = right, A = absent, B = bilateral, LPM = left lateral plate mesoderm.

### Gpr22 regulates LR asymmetry through its function in the KV

**Figure 3:**
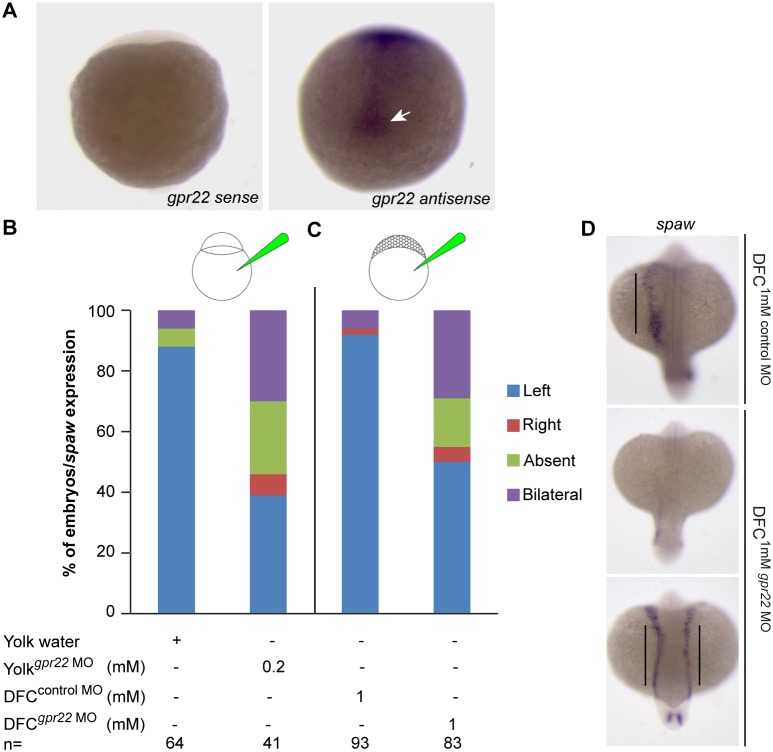
Gpr22 regulates LR asymmetry through its function in the KV. (**A**) WISH on 1–5 somite stage zebrafish embryos using the *gpr22* sense or *gpr22* antisense probe. Shown are the dorsal views of the tail bud region with anterior to the top. *gpr22* is expressed in the axial structures and in the forming KV *(arrow)*. (**B, C**) Percentage of embryos with randomized *southpaw (spaw)* expression, injected with (**B**) water or 0.2 mM *gpr22* MO into the yolk at one cell stage (knock down in the whole embryo), or with (**C**) 1 mM control MO or 1 mM *gpr22* MO into the yolk at mid-blastula stage (DFC/KV specific knock down). A DFC/KV specific knock down of *gpr22* results in a similar percentage of zebrafish embryos showing randomized *southpaw* expression as a knock down in the whole embryo. (**D**) Representative images of panel (**C**). MO = morpholino, n = number of analyzed embryos, yolk^superscript^ = injected at one cell stage, DFC^superscript^ = injected at mid-blastula stage, WISH = whole-mount in situ hybridization, DFC = dorsal forerunner cell, LR = left-right, KV = Kupffer’s vesicle.

Induction of LR asymmetry in zebrafish depends on the rotational movement of the KV cilia to create a directional fluid flow [24], [30]. To investigate whether Gpr22 functions in the KV, we first analyzed the expression pattern of *gpr22* during zebrafish and KV development, using two different *gpr22* antisense probes and a control sense probe. During early stages of development, *gpr22* was expressed ubiquitously (**Fig. S5A**). From bud stage onwards, the expression pattern became restricted to the axial structures and the developing KV (**Fig. 3A** **and Fig. S5B, B’**). In contrast, we did not observe any signal in embryos treated with the control sense probe (**Fig. 3A**). The expression pattern of *gpr22* at later stages of development (20 somite stage until long-pec) was previously described and reproduced by us (**Fig. S5C–D’**) (www.zfin.org) [31]. To investigate the KV-specific function of Gpr22 further, we performed a DFC/KV specific knock down of *gpr22* by the injection of *gpr22* MO into the yolk at the mid-blastula stage (3–4 hpf) (**Fig. 3C**), as previously described [29]. At this stage, the cytoplasmic bridges between the yolk and the embryo are closed, except for the ones between the yolk and the DFCs. Consequently, the MO diffuses only into the DFCs without affecting other embryonic cells. All the DFC/KV specific knock downs (injections at 1000-cell stage) were performed by injecting fluorescein-tagged control MO instead of water, or fluorescein-tagged *gpr22* MO. The fluorescence allowed us to select embryos at 1–5 somite stage in which only the DFCs were hit [32]. The control MO was available in the lab and designed to target the 5′UTR of *dus4l* mRNA. However, injection of this MO in zebrafish embryos at one cell stage or mid-blastula stage did not induce phenotype and more specifically, did not cause left-right asymmetry defects (**Fig. 3C, D**). Therefore, we considered the MO as a valid control. Since the MOs often aggregated at the site of injection and did not diffuse into the DFCs, we increased the injected MO dose to 1 mM. A WISH for the LR marker *southpaw* at the 20 somite stage revealed that a KV-specific knock down of *gpr22* induced randomized expression of *southpaw*, identical to the phenotype of a knock down in the whole embryo (**Fig. 3B–D**). These data indicate that Gpr22 regulates LR patterning through its specific function in the KV and not elsewhere.

### Gpr22 affects cilia length and structure in the KV

**Figure 4:**
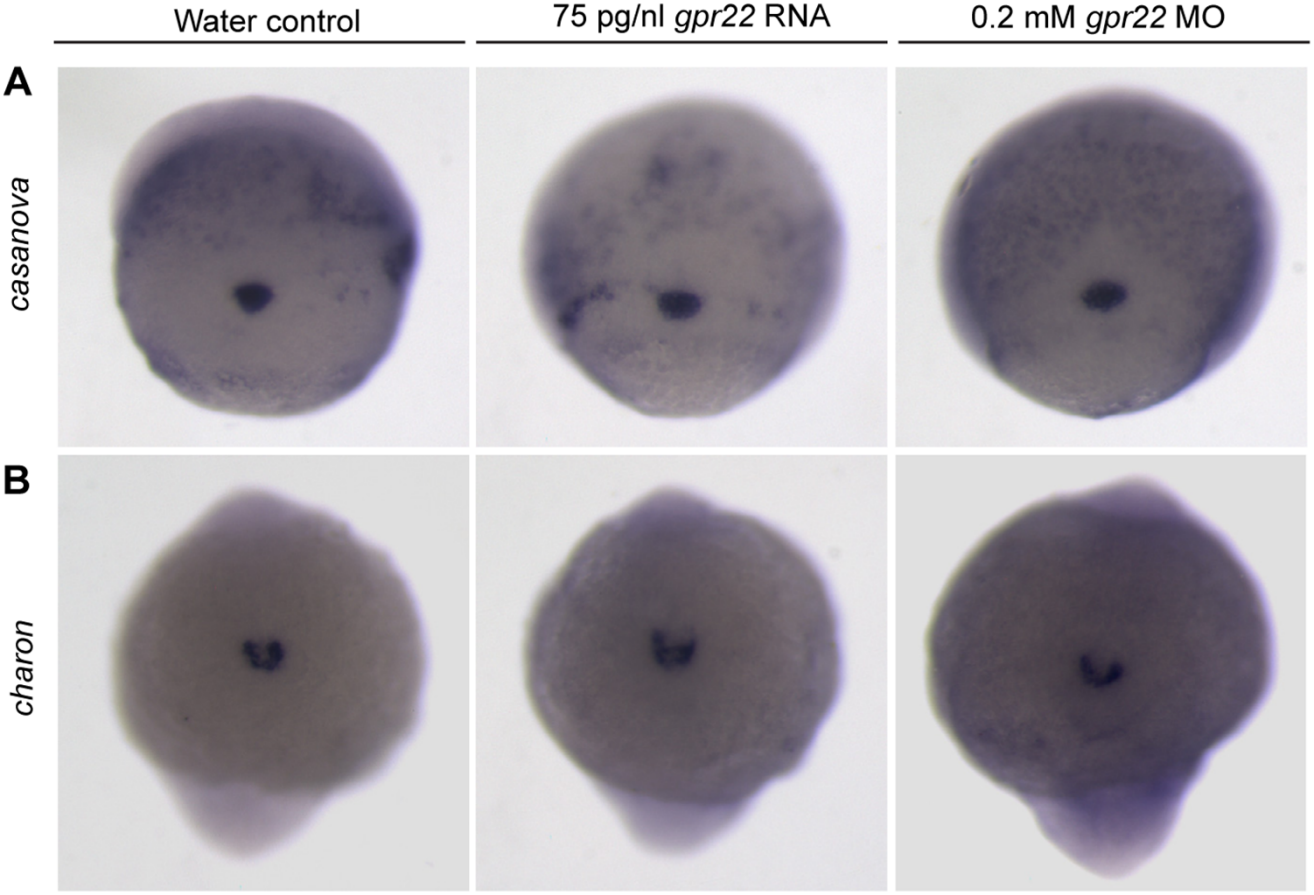
Gpr22 is not involved in KV formation or morphology. (**A**) WISH for the DFC marker *casanova* on embryos at 75% epiboly or (**B**) for the KV marker *charon* on 10 somite stage embryos. Shown is (**A**) the dorsal view (**B**) of the tailbud region with anterior to the bottom. *gpr22* overexpression or knock down does not alter the expression of *casanova* or *charon*. MO = morpholino, KV = Kupffer’s vesicle, WISH = whole mount in situ hybridization, DFCs = dorsal forerunner cells.

**Figure 5:**
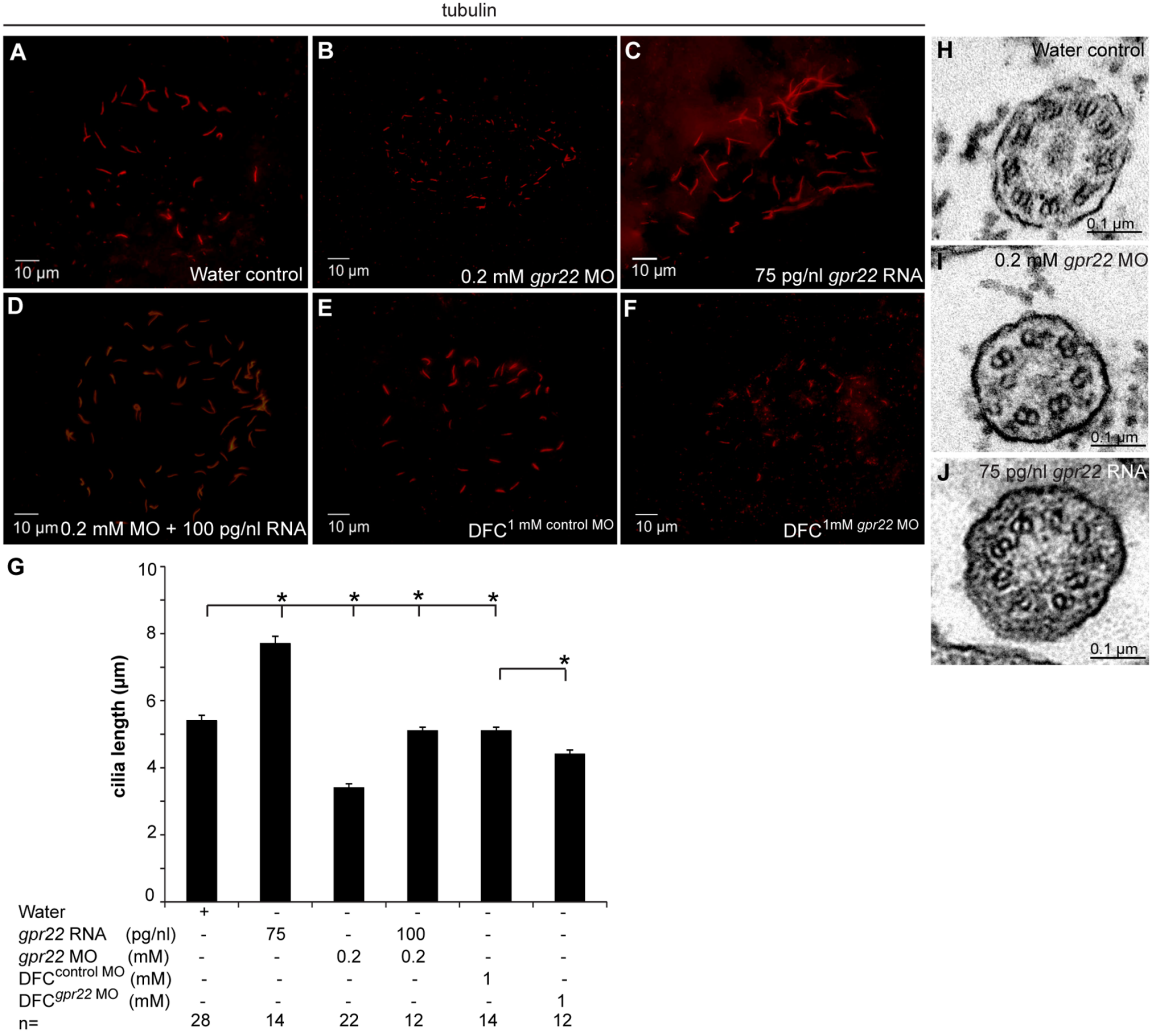
Gpr22 affects cilia length and structure in the KV. (**A–F**) Immunostaining with anti-acetylated tubulin antibody on embryos at 10 somite stage injected at (**A–D**) one cell stage with (**A**) water, (**B**) 0.2 mM *gpr22* MO, (**C**) 75 pg/nl *gpr22* RNA, (**D**) 0.2 mM *gpr22* MO and 100 pg/nl *gpr22* RNA, or injected at (**E, F**) mid-blastula stage with (**E**) 1 mM control MO, (**F**) 1 mM *gpr22* MO. Shown are the cilia of the KV. (**G**) Quantification of average cilia length ± s.d. (**B, G**) Whole embryo or (F, G) DFC-specific knock down of *gpr22* significantly reduces cilia length. (**C, G**) In contrast, *gpr22* overexpression results in significantly longer cilia. (**D, G**) Co-injection of 0.2 mM MO and 100 pg/nl RNA almost completely rescues cilia length. (**H–J**) TEM of cross sections of the KV cilia at 10 somite stage. (**H**) Structure of water control KV cilia showing 9 parallel outer microtubule doublets. (**I**) *gpr22* knock down or (**J**) overexpression results in a disruption of the proper microtubuli arrangement or (**I**) even an absence of doublets. MO = morpholino, n = number of analyzed embryos, DFC = dorsal forerunner cells, DFC^superscript^ = injected at mid-blastula stage, KV = Kupffer’s vesicle, TEM = transmission electron microscopy, * = P<0.01 (unpaired, two-tailed T-test).

Since it is possible that *gpr22* deregulation affects KV formation or morphology, we investigated the early steps of KV formation. It has been shown that the assembly of the DFCs is essential in the formation of a functional KV [33]. The DFC were visualized by a WISH for the marker *casanova* at 75%–85% epiboly [34],[35]. However, the assembly of the DFCs was not affected in embryos injected with *gpr22* mRNA or MO (**Fig. 4A**). Next, we investigated KV morphology using a WISH for *charon* at the 10 somite stage. *charon* is expressed in the cells lining the KV and is a commonly used marker to study KV morphology [36], [37]. *charon* expression was not altered in embryos with deregulated *gpr22* expression (**Fig. 4B**). These data support our notion that Gpr22 is not involved in KV formation or morphology. Another possibility is that Gpr22 is involved in KV ciliogenesis. It has been shown that the disruption of KV cilia number, length or structure leads to a loss of the directional fluid flow, a disturbed expression of the left-specific LPM genes and consequently defective LR patterning [24], [30]. Immunostaining with anti-acetylated tubulin antibody at 10 somite stage revealed that *gpr22* morphants had significantly shorter KV cilia, compared to water injected controls (3.4±0.12 µm instead of 5.4±0.16 µm; P = <0.01) (**Fig. 5A, B, G**). In contrast, overexpression of *gpr22* resulted in significantly longer cilia (7.7±0.22 µm instead of 5.4±0.16 µm; P = <0.01) (**Fig. 5:** **A, C, G**). Moreover, the co-injection of 0.2 mM *gpr22* MO and 100 pg/nl *gpr22* RNA at one cell stage almost completely rescued cilia length (5.1±0.11 µm instead of 5.4±0.16 µm; P = <0.01) (**Fig. 5D, G**). Importantly, a DFC-specific knock down of *gpr22* also resulted in significantly shorter cilia, compared to embryos injected at mid-blastula stage with control MO (4.4±0.13 µm instead of 5.1±0.11 µm; P = <0.01) (**Fig. 5E–G**). We noticed that the injection of the control MO at mid-blastula stage caused a very mild reduction in cilia length, compared to the injection of water at one cell stage (5.1±0.11 µm instead of 5.4±0.16 µm; P = <0.01) (**Fig. 5A, E, G**). However, this had no consequence on the expression of *southpaw* and did not induce LR asymmetry defects (**Fig. 3C, D**). We did not observe a detectable change in cilia number in all conditions tested. Next, we investigated whether the structure of the KV cilia was affected in embryos with deregulated *gpr22* expression. Therefore, we performed a TEM ultra-structural analysis on cross sections of the KV cilia at 10 somite stage. Control cilia showed the typical 9 parallel outer microtubule doublets (**Fig. 5H**). In contrast, *gpr22* overexpression or knock down resulted in a disruption of the proper microtubule arrangement or even the absence of doublets (**Fig. 5I, J**). Our data suggest that deregulation of *gpr22* expression results in defective KV cilia length and structure, leading to LR asymmetry defects.

### Gpr22 does not act upstream of the cilia master regulators Foxj1a and Rfx2

**Figure 6:**
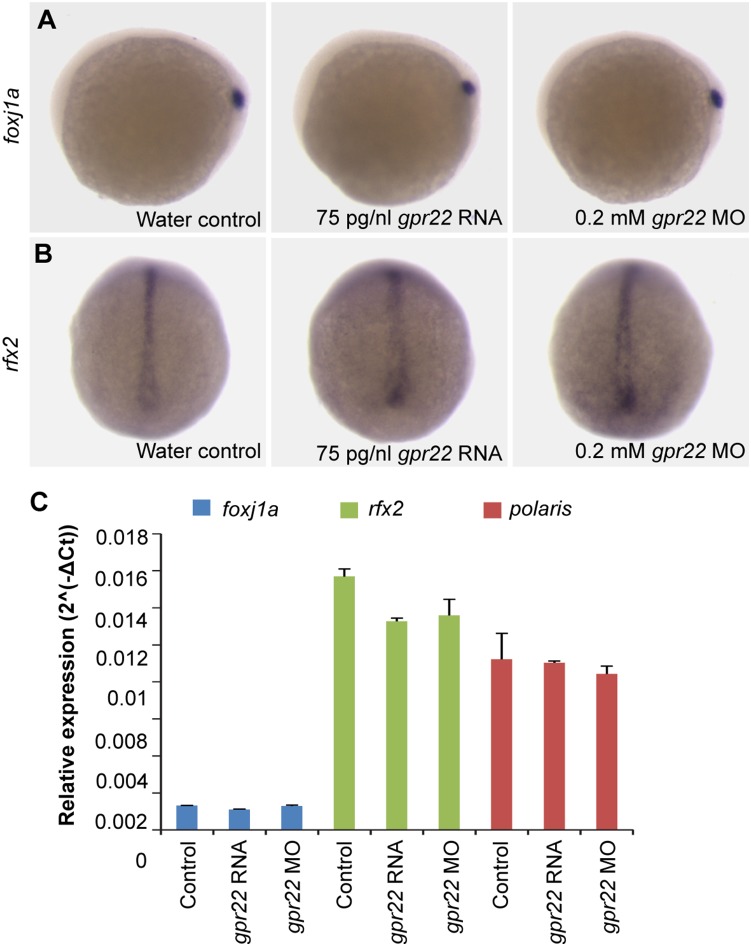
Gpr22 does not act upstream of Foxj1a/Rfx2 in KV ciliogenesis. (**A, B**) WISH of (**A**) *foxj1a* or (**B**) *rfx2* on embryos at bud stage. Shown are (**A**) the lateral view with anterior to the left or (**B**) dorsal view with anterior to the top. *gpr22* knock down or overexpression does not alter the DFC expression of *foxj1a* or *rfx2*, two master regulators of ciliogenesis. (**C**) Confirmation by qPCR of the *foxj1a*, *rfx2* and *polaris* (target gene) RNA levels in the whole embryo, normalized to *beta-actin*. MO = morpholino, WISH = whole-mount in situ hybridization, DFC = dorsal forerunner cell, KV = Kupffer’s vesicle.

To investigate whether Gpr22 affects KV ciliogenesis by regulating the expression of *foxj1a* or *rfx2*, we carried out WISH and qPCR for these transcription factors on bud stage embryos. There were no detectable differences in the expression pattern or level (**Fig. 6A, B**) of these genes in LOF or GOF embryos. Furthermore, the expression level of a *foxj1a/rfx2* target gene, *polaris*, was also unchanged, indicating that changes in *gpr22* expression did not result in changes of the transcriptional activity of *foxj1a* or *rfx2* (**Fig. 6C**). Thus, Gpr22 does not affect KV ciliogenesis by changing the expression or transcriptional activity of *foxj1a* or *rfx2.*

## Discussion

We found that Gpr22 affects KV cilia length and structure during zebrafish development. Overexpression or knock down of *gpr22* in the zebrafish embryo resulted in a downwards curvature of the tail, heart edema and LR patterning defects. These phenotypes are frequently observed in zebrafish with mutations in genes involved in ciliogenesis [22]. Remarkably, zebrafish ciliary mutants all show the same range of phenotypes, independently on which gene is mutated or which step of ciliogenesis is affected. For example, the *fleer* mutant has a mutation in a gene involved in the post-translational polyglutamylation of tubulin and consequently has defects in cilia structure [38]. The *oval* and *ift88* (*polaris*) mutants have a defective intraflagellar transport system resulting in changes in cilia length and structure [39], [40]. In the *shmalhans* mutant, cilia motility is affected due to a destabilization of the dynein arms [41]. Nonetheless, they are all characterized by a bent tail, heart edema, hydrocephalus, kidney cysts, otolith defects and randomized LR patterning. Besides the curved tail, the heart edema and the LR patterning defects, embryos with deregulated *gpr22* expression also displayed otolith defects, suggesting that Gpr22 affects ciliogenesis in other organs besides the KV (**Fig. S6**) [42].

Any defects in KV cilia length or structure will lead to a disturbance of the directional fluid flow and thus randomized LR patterning, which can easily be used as a read-out for changes in KV ciliogenesis [24], [30]. Indeed, the randomized LR patterning defect observed in both *gpr22* morphants and embryos with an overexpression of *gpr22*, was the result of shortening or lengthening, respectively, of the KV cilia and changes in the ultrastructure of the cilia.

We noticed that a DFC/KV specific knock down of *gpr22* results in a milder reduction of KV cilia length (4.4±0.13 µm instead of 5.1±0.11 µm; P = <0.01), compared to a knock down in the whole embryo (3.4±0.12 µm instead of 5.4±0.16 µm; P = <0.01). Although we attempted to solve this issue by increasing the injected dose, it still might be that the MO did not diffuse efficiently into the DFCs, as it often aggregates at the site of injection. Another possible explanation is that an amount of functional Gpr22 protein is already present at the mid-blastula stage, which can partially compensate for the MO-blocked translation of the *gpr22* RNA.

In contrast to the zebrafish *gpr22* morphants, *Gpr22* null mice display no obvious defects. However, the function of *Gpr22* in ciliogenesis has not been specifically investigated in this study [7]. Further, it has been previously reported that there can be differences between zebrafish and mice in the requirement of cilia proteins. For example, a knock down of *septin 6* or *lethal giant larvae 2* results in cilia-associated phenotypes in zebrafish, while the mouse knock outs lack any phenotype [43], [44]. Additionally, GPCRs form oligomeric complexes, making it difficult to predict the outcome of a specific GPCR knock down and raising the possibility that GPCRs can function redundantly [45], [46].

Many of the characterized pathways such as FGF, Wnt or Notch, regulate KV cilia length in zebrafish by regulating the master regulators of ciliogenesis, Foxj1 or Rfx2 [47], [48]. Our data indicate, however, that Gpr22 does not alter the expression or transcriptional activity of neither of these transcription factors. It has been shown that overexpression of GPR22 in HEK-293 cells led to the inhibition of PKA activity [7] and that a decrease in PKA activity inhibits ciliogenesis and reduces cilia length [49]–[51]. However, embryos with an overexpression of *gpr22*, which should have a decrease in PKA activity, have the opposite phenotype. Thus, based on the literature, it is unlikely that Gpr22 signals in a PKA-depending manner to regulate KV cilia length.

Our finding that Gpr22 is involved in ciliogenesis, is supported by many studies that point towards a function of other GPCRs in ciliogenesis. GPCRs are often targeted to the cilia, such as the rhodopsin receptor in the outer segment of photoreceptors, the smoothened receptor controlling Hedgehog signaling in the primary cilia and the serotonin type 6 and somatostatin type 3 receptors in the neuronal cilia [12], [52], [53]. Further, it has been shown that the localization and trafficking of GPCRs in the neuronal cilia is regulated by the ciliary Bardet-Biedl syndrome proteins [54]. Additionally, conserved ciliary localization sequences have been found within GPCRs, like the AxxxQ motif in the IL3 and the VxPx motifs [12], [52], [55]. Finally, a chemical screen performed in *Chlamydomonas* identified the class A GPCRs as regulators of ciliogenesis [13]. The lack of a zebrafish Gpr22 antibody, however, prevented us from investigating the subcellular localization of the Gpr22 protein in zebrafish, but we were able to detect porcine Gpr22 in cilia protein extracts isolated from a porcine kidney epithelial cell line (CL4) (in collaboration with Wim Annaert and Applonia Rose, Laboratory for Membrane Transport, University of Leuven). Moreover, analysis of the Gpr22 protein sequence for cilia targeting motifs, revealed two VxPx and one AxxxQ motif, suggesting that Gpr22 might be targeted to the cilia. Nonetheless, many regulators of ciliogenesis are not targeted to the cilium per se, such as transcription factors, miRNAs, proteins associated with the centrosome, the basal bodies, actin etc. [25], [56]–[59].

Despite the growing list of genes and pathways affecting ciliogenesis, including Gpr22, the actual mechanism of maintaining cilia length and structure is still poorly investigated. Only theoretical models have been proposed, like the ‘balance-point’ mechanism [17]. In this model, the efficiency of the intraflagellar transport (IFT) is reversely proportional to the cilia length. Since the amount and distribution of IFT trains is the same in longer and shorter cilia, longer cilia will have smaller IFT trains that have to carry cargo along a longer distance. This results in the transport of less cargo to the tip and a net disassembly of the cilium. The steady-state length is reached when the assembly rate is the same as the disassembly rate. The question still remains how the amount of IFT particles is maintained in the cilium. Gpr22, together with genes already identified in screens for cilia length mutants, might control IFT quantity and therefore, cilia length and structure [17].

*GPR22* is part of a gene cluster that has been genetically associated with human OA. Initial analysis revealed that *GPR22* mRNA is present in both monolayer cultures (2D) and in pellets (3D) of human articular chondrocytes, indicating that *GPR22* is also expressed in an environment that more accurately recapitulates articular cartilage. OA mouse models displayed expression of GPR22 in the articular cartilage of the knee joints [8], [9]. However, the function of GPR22 in the disease process is still controversial, since Raine et al. could not detect *GPR22* RNA expression in human cartilage or joint tissues [8]–[10]. Nevertheless, our research could give more insight into the possible role of GPR22 in OA. Cilia are indeed necessary for proper cartilage/bone development and maintenance, mainly because of their essential role in Hedgehog, Wnt and mechano/chemosensing signaling pathways [60], [61]. It has been proposed that the primary cilia of chondrocytes act to maintain the columnar organization of the growth plate by a process termed chondrocyte rotation [62], [63]. The authors found that a conditional knock out of the IFT protein Kif3a in the chondrocytes of mice embryos, resulting in a lack of primary chondrocyte cilia, led to a disrupted columnar orientation in the growth plate and post-natal dwarfism [63]. Further, human ciliopathies are often characterized by skeletal defects, such as the Bardet-Biedl syndrome, the short rib-polydactyly group (SRPs) of ciliopathies and the Jeune syndrome (ATD) [61]. Moreover, *Col2aCre;Ift88fl/fl* mice, in which the primary cilia of the chondrocytes are lost, displayed symptoms of early OA because of a disturbed balance in the HH signaling pathway [64]. Additionally, it has been shown that the incidence and length of primary chondrocyte cilia increased at the degenerative articular surface in osteoarthritic tissue [65].

To conclude, our research identifies a novel player in the field of ciliogenesis and might open the door to new insights in the potential function of GPR22 in OA.

## Materials and Methods

### Zebrafish strains and maintenance

We confirm that all animal experiments were done according to the latest regulations set by the Belgian authorities that follow the European Parliament and Council Directive 2010/63/EU on the protection of animals used for scientific purposes. We confirm we also obtained approval by the Animal Care Committee of KU Leuven, the acting Institutional Animal Care and Use Committee (IACUC) of KU Leuven.

Adult zebrafish (*Danio rerio*) were maintained at 28.5°C, on a 14/10 hour, light/dark cycle under standard aquaculture conditions as described (www.zfin.org). All experiments were performed using embryos obtained from random matings of the wild type Oregon AB strain or the *Tg(cmlc2:GFP)* strain [29]. Embryos were staged by hours post-fertilization (hpf) and the number of somites according to Kimmel et al. [66].

### Morpholino (MO) microinjections

Fluorescein tagged antisense MO oligonucleotides targeting the 5′UTR of *gpr22* RNA and a control MO were obtained from Gene Tools, LLC (MO1: 5′-TCCCCTCCCTTGTGTTCCCTGTCCT-3′; MO2: 5′-ACTCCATCCATACTCCAATGCCTCT-3′; control MO: 5′-GGATTAAAATCCGCTACTCACATCC-3′; p53 MO: 5′-GCGCCATTGCTTTGCAAGAATTG-3′). Unless specified otherwise in the Results, 0.2 mM MO1 together with 0.1 mM p53 MO, or 0.4 mM MO2 were injected into the yolk of one cell stage embryos in a volume of 1 nl. To target MOs specifically to the dorsal forerunner cells (DFCs), 1 mM of MO was injected into the yolk at mid-blastula stage as previously described [32]. After injection, embryos with fluorescent-MO positive DFCs were selected at the 1–5 somite stage for further analysis.

### Cloning and mRNA microinjections

The protein conservation between zebrafish and human GPR22 was determined by the program “specialized blast”, “align” (www.ncbi.nlm.nih.gov/BLAST/). We identified the 7TM domains using the hidden Markov model (TMHMM Server v. 2.0, http://www.cbs.dtu.dk/services/TMHMM/) [67]. Zebrafish cDNA was generated using the RevertAid First Strand cDNA Synthesis Kit (Fermentas) from total RNA extracted from 24-hpf wild-type embryos by the High Pure RNA Tissue Kit (Roche). Next, we used the generated cDNA as a template to amplify the full-length open reading frame of zebrafish *gpr22.* The resulting fragments were then cloned into the mammalian expression vector *pCS2+* and sent out for sequence analysis to confirm the identity. Capped mRNA was synthesized from the *pCS2+* plasmid using the mMESSAGE mMACHINE SP6 Kit (Ambion). Unless specified otherwise in the Results, overexpression studies were performed by injecting 75 pg/nl of *gpr22* mRNA into the yolk of one cell stage embryos in a volume of 1 nl.

### Whole mount in situ hybridization (WISH)

WISH was performed as described previously [68]. RNA antisense or sense probes were generated from linearized plasmids using SP6, T3 or T7 polymerase and a digoxigenin labeling mix (Roche).

### Whole mount immunostaining

Embryos were fixed in 4% PFA or Dent’s fixative (80% methanol: 20% dimethyl sulfoxide) and kept ON at 4°C. Subsequently, we followed the protocol described previously for immunostaining [69]. Cilia were stained using monoclonal anti-acetylated tubulin antibody (Sigma) in a 1/1000 dilution. Alexa Fluor 555 Goat Anti-mouse (Life Technologies) was used as a secondary antibody in a 1/1000 dilution. Pictures from the KV cilia were obtained with a Leica DMR fluorescence microscope (Leica Microsystems) after flatmounting the embryos on glass slides in 90% glycerol and cilia length was measured by ImageJ software. Differences in average cilia length between conditions were evaluated using the two-tailed unpaired Student’s *t*-test. Results were considered to be significant when P<0.01.

### Transmission electron microscopy (TEM)

Embryos were fixed in 0.1 M sodium-cacodylate buffer containing 2.5% glutaraldehyde and 2% PFA ON at 4°C. They were additionally fixed in 1% osmium tetroxide for 2 h at 4°C. After dehydration by ethanol series, we performed an ‘en bloc’ staining in 4% uranyl acetate for 30 min at 4°C. Subsequently, embryos were treated with propylene oxide for 2×15 min and embedded in 100% epoxy resin for 2 d at 60°C. 70 nm sections of the KV were post stained with 4% uranyl acetate and lead citrate before analysis with a JEM-2100 JEOL microscope.

### qPCR

Total RNA was isolated and pooled from 40 deyolked bud stage embryos using the High Pure RNA Isolation Kit (Roche) and cDNA was synthesized according to the manufacturer’s protocol (Primescript, Takara). qPCR was performed with gene specific intron spanning primers using the SYBR Premix Ex Taq I kit (Takara). RNA levels were normalized to the housekeeping gene *beta-actin*.

## Supporting Information

Figure S1 ***gpr22*** **knock down results in a dose-dependent curvature of the tail. (A–C)** Lateral views with anterior to the left of zebrafish embryos at 24 hpf, injected at one cell stage with **(A)** water, **(B)** 0.2 mM *gpr22* MO1 with 0.1 mM p53 MO or **(C)** without p53 MO. **(D)** Quantification of the tail phenotypes. **(A, B, D)** Knock down of *gpr22* with either MO1 or MO2, results in a dose-dependent WT, mild, medium or severe downwards curvature of the tail. **(B, C)** Co-injection with p53 MO rescues the head necrosis caused by MO1. MO = morpholino, hpf = hours post-fertilization, n = number of analyzed embryos, WT = wild type.
(TIF)

Figure S2 ***gpr22*** **overexpression results in a dose-dependent curvature of the tail. (A–C)** Lateral views with anterior to the left of zebrafish embryos at 24 hpf, injected at one cell stage with **(A)** water or **(B)** 75 pg/nl *gpr22* RNA. **(C)** Quantification of the tail phenotypes. **(A–C)** Overexpression of *gpr22* results in a dose-dependent WT, mild, medium or severe curvature of the tail. hpf = hours post-fertilization, n = number of analyzed embryos, WT = wild type.
(TIF)

Figure S3 ***gpr22*** **knock down results in randomized expression of the left-specific LPM markers.** **(A, B)** Dorsal views with anterior to the top of embryos at the 20 somite stage. **(A)** WISH for *lefty1* or **(B)** *pitx2a*. The expression of the LR markers *lefty1* and *pitx2a* is randomized in *gpr22* morphants *(straight line)*. LPM = lateral plate mesoderm, WISH = whole mount in situ hybridization.MO = morpholino.
(TIF)

Figure S4 ***gpr22*** **overexpression results in defective LR patterning.** **(A)** Ventral or **(B, C)** dorsal views with anterior to the top of embryos at **(A, B)** 4 dpf or **(C)** 20 somite stage. **(A)** Transgenic embryos expressing GFP under control of the *cmlc2* promoter. *gpr22* overexpression results in (from left to right) normal, absent, reversed or bicardial cardiac looping (not shown). **(B)** WISH for *foxa3*. The liver of water injected control embryos develops at the left side of the embryo. In contrast, the position of the liver in embryos injected with *gpr22* RNA is randomized *(straight line)*. **(C)** WISH for *southpaw (spaw)*. The expression of the early LR marker *southpaw* is randomized in *gpr22* injected embryos *(straight line)*. V = ventricle, A = atrium, dpf = days post-fertilization, GFP = green fluorescence protein, *cmlc2* = *cardiac myosin light chain type 2*, LR = left-right, WISH = whole mount in situ hybridization.
(TIF)

Figure S5 **Expression analysis of** ***gpr22*** **in zebrafish development.** **(A–D’)** WISH for *gpr22*. **(A, B, C, D)** Lateral view with anterior to the left. **(B’)** Dorsal view of the tail bud region. **(C’)** Anterior view. **(D’)** Dorsal view with anterior to the top. **(A, B)** From shield to bud stage, *gpr22* is ubiquitously expressed. **(B’)** From bud stage onwards, the expression pattern becomes more and more restricted to the axial structures and the developing KV *(arrow)*. **(C–D’)** At later stages, *gpr22* is expressed in several brain regions, the heart and some ciliated sensory organs, like **(D)** the retina and **(D, D’)** the nasal sac. WISH = whole-mount in situ hybridization, KV = Kupffer’s vesicle, hpf = hours post-fertilization, r = retina, ns = nasal sac.
(TIF)

Figure S6 ***gpr22*** **morphants display otolith defects.** Left ear with anterior to the left of zebrafish embryos at 2 dpf, injected at one cell stage with **(A)** water or **(B, C)** 0.2 mM *gpr22* MO. **(A)** Control embryos have 2 tethered otoliths *(white asterisks)* at the anterior and posterior poles of the otic vesicle. In contrast, *gpr22* morphants display **(B)** fused otoliths or **(C)** an increased number of otoliths, which are not correctly positioned. dpf = days post-fertilization, MO = morpholino.
(TIF)
