# Supplementary figures and images for: Orphan G-Protein Coupled Receptor 22 (Gpr22) Regulates Cilia Length and Structure in the Zebrafish Kupffer’s Vesicle
Source: PLoS One. 2014 Oct 21;9(10):e110484. doi: 10.1371/journal.pone.0110484 (PMC4204907; doi:10.1371/journal.pone.0110484)

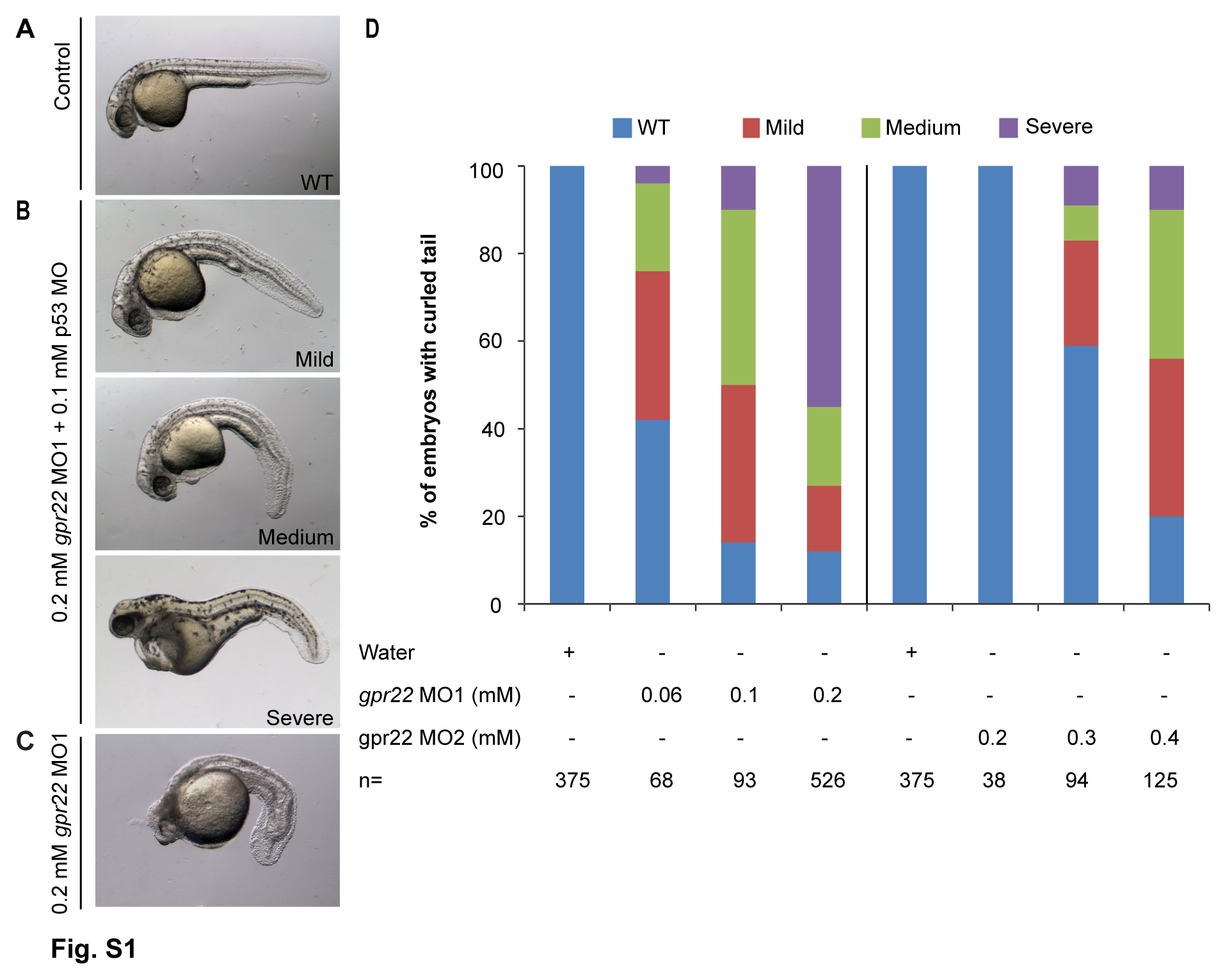

Supplement: Figure S1 — gpr22 knock down results in a dose-dependent curvature of the tail. (A–C) Lateral views with anterior to the left of zebrafish embryos at 24 hpf, injected at one cell stage with (A) water, (B) 0.2 mM gpr22 MO1 with 0.1 mM p53 MO or (C) without p53 MO. (D) Quantification of the tail phenotypes. (A, B, D) Knock down of gpr22 with either MO1 or MO2, results in a dose-dependent WT, mild, medium or severe downwards curvature of the tail. (B, C) Co-injection with p53 MO rescues the head necrosis caused by MO1. MO = morpholino, hpf = hours post-fertilization, n = number of analyzed embryos, WT = wild type. (TIF) [file pone.0110484.s001.tif]

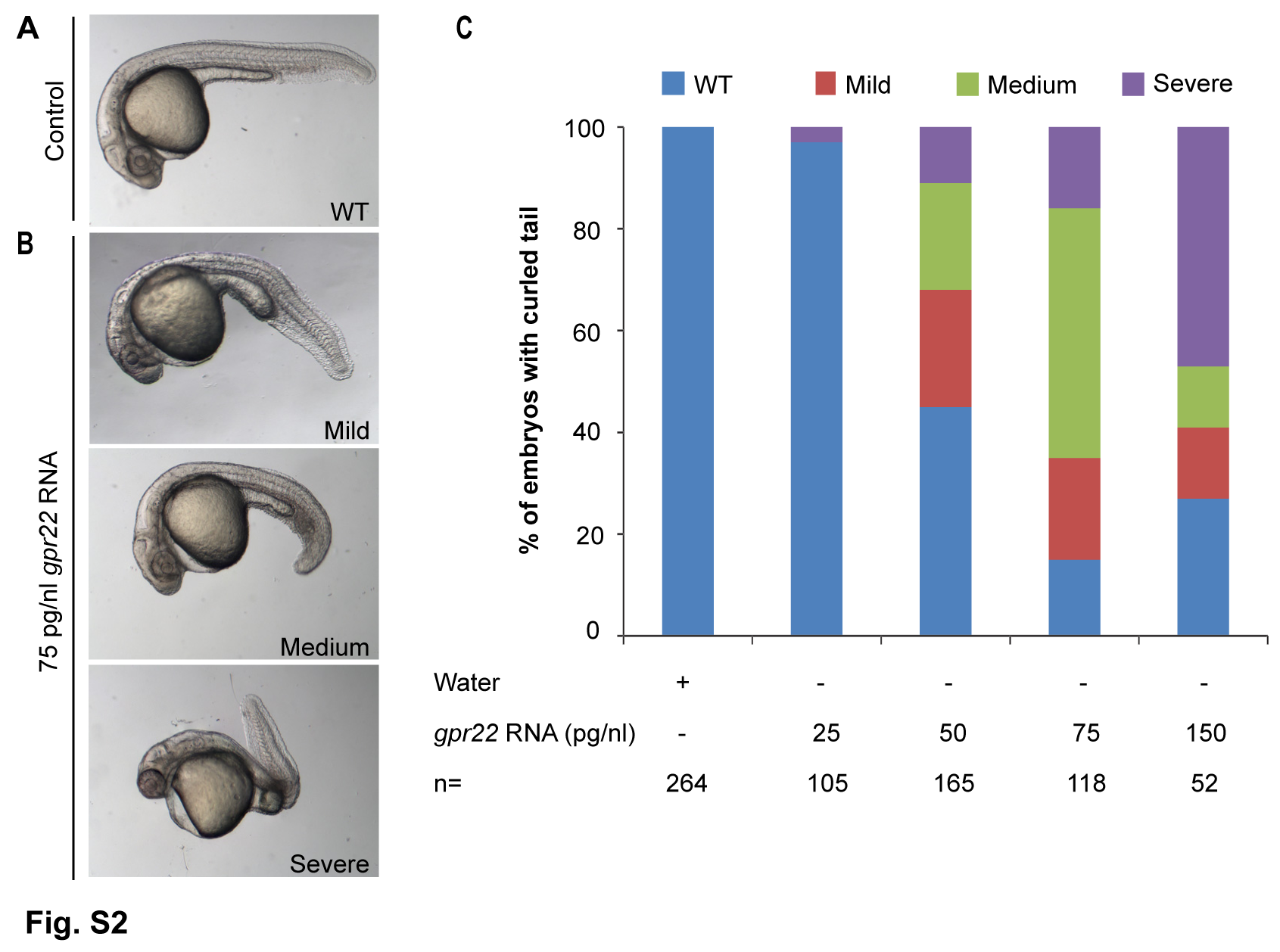

Supplement: Figure S2 — gpr22 overexpression results in a dose-dependent curvature of the tail. (A–C) Lateral views with anterior to the left of zebrafish embryos at 24 hpf, injected at one cell stage with (A) water or (B) 75 pg/nl gpr22 RNA. (C) Quantification of the tail phenotypes. (A–C) Overexpression of gpr22 results in a dose-dependent WT, mild, medium or severe curvature of the tail. hpf = hours post-fertilization, n = number of analyzed embryos, WT = wild type. (TIF) [file pone.0110484.s002.tif]

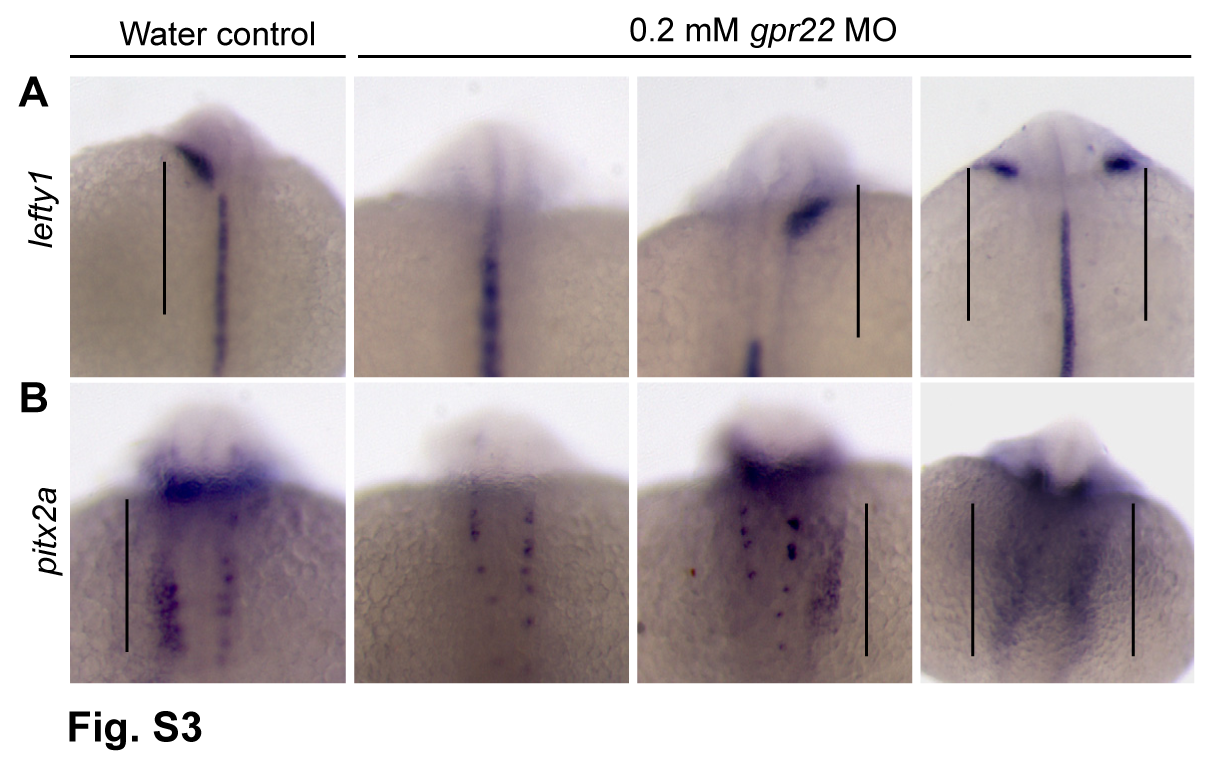

Supplement: Figure S3 — gpr22 knock down results in randomized expression of the left-specific LPM markers. (A, B) Dorsal views with anterior to the top of embryos at the 20 somite stage. (A) WISH for lefty1 or (B) pitx2a. The expression of the LR markers lefty1 and pitx2a is randomized in gpr22 morphants (straight line). LPM = lateral plate mesoderm, WISH = whole mount in situ hybridization.MO = morpholino. (TIF) [file pone.0110484.s003.tif]

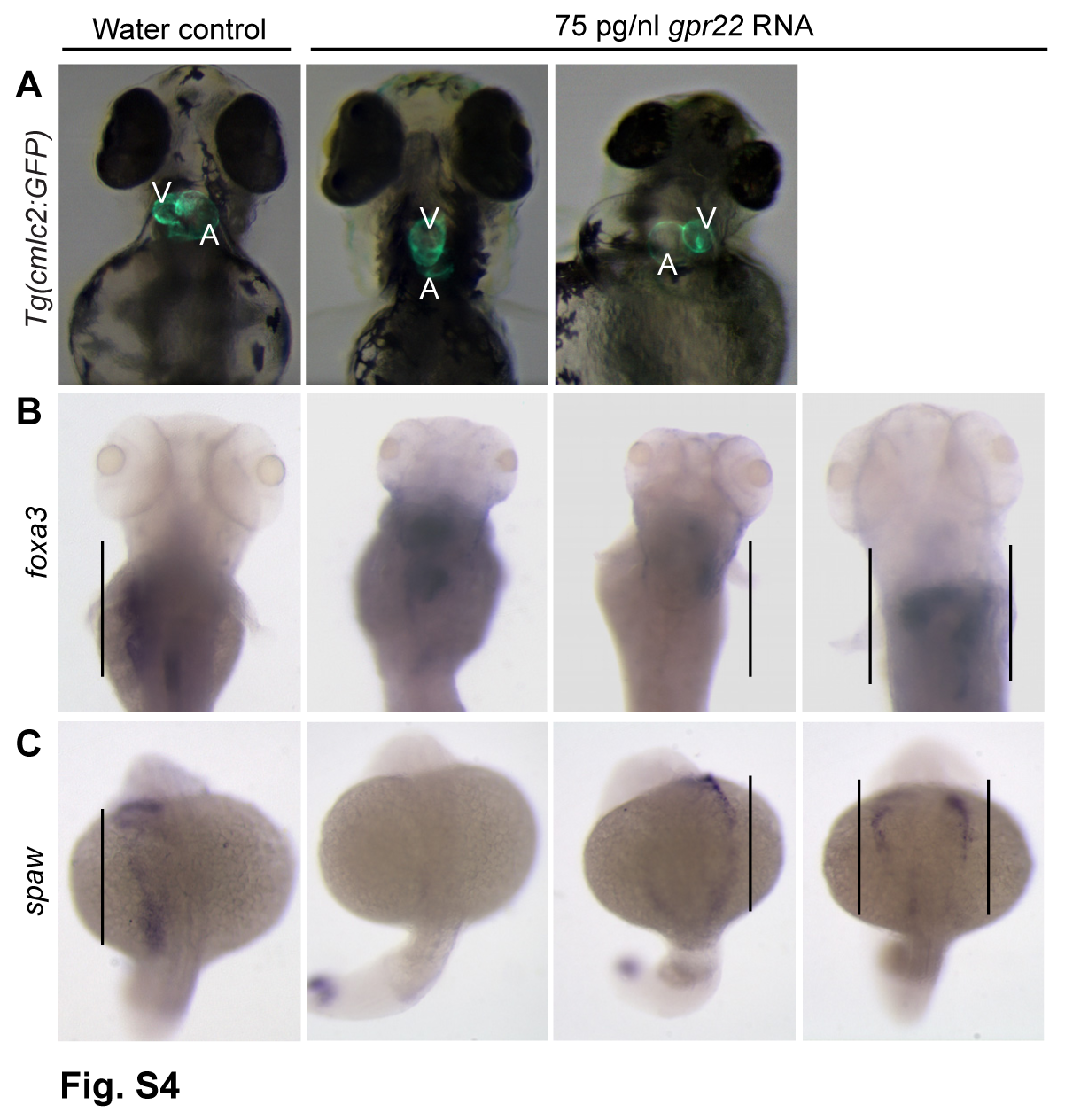

Supplement: Figure S4 — gpr22 overexpression results in defective LR patterning. (A) Ventral or (B, C) dorsal views with anterior to the top of embryos at (A, B) 4 dpf or (C) 20 somite stage. (A) Transgenic embryos expressing GFP under control of the cmlc2 promoter. gpr22 overexpression results in (from left to right) normal, absent, reversed or bicardial cardiac looping (not shown). (B) WISH for foxa3. The liver of water injected control embryos develops at the left side of the embryo. In contrast, the position of the liver in embryos injected with gpr22 RNA is randomized (straight line). (C) WISH for southpaw (spaw). The expression of the early LR marker southpaw is randomized in gpr22 injected embryos (straight line). V = ventricle, A = atrium, dpf = days post-fertilization, GFP = green fluorescence protein, cmlc2 = cardiac myosin light chain type 2, LR = left-right, WISH = whole mount in situ hybridization. (TIF) [file pone.0110484.s004.tif]

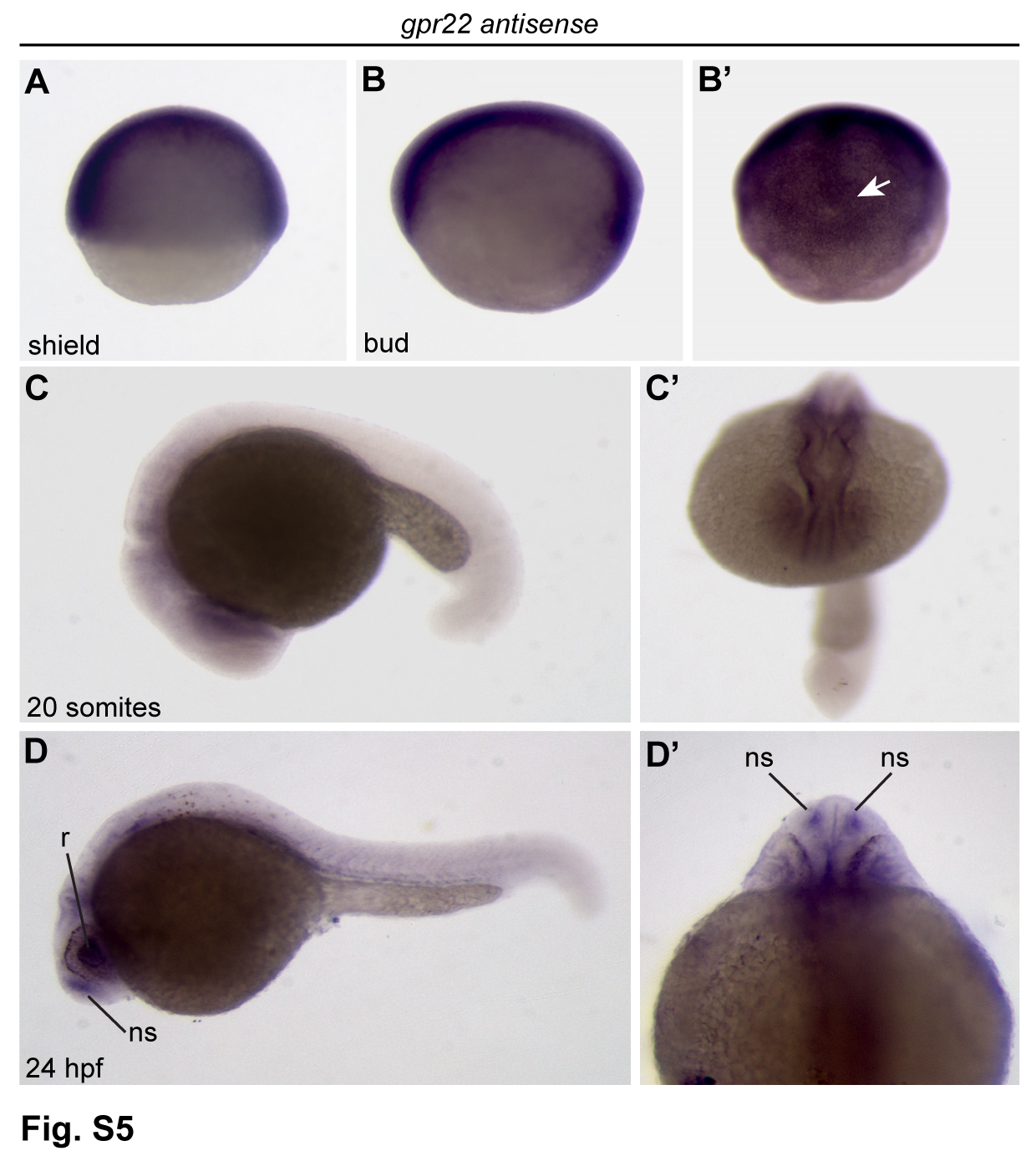

Supplement: Figure S5 — Expression analysis of gpr22 in zebrafish development. (A–D’) WISH for gpr22. (A, B, C, D) Lateral view with anterior to the left. (B’) Dorsal view of the tail bud region. (C’) Anterior view. (D’) Dorsal view with anterior to the top. (A, B) From shield to bud stage, gpr22 is ubiquitously expressed. (B’) From bud stage onwards, the expression pattern becomes more and more restricted to the axial structures and the developing KV (arrow). (C–D’) At later stages, gpr22 is expressed in several brain regions, the heart and some ciliated sensory organs, like (D) the retina and (D, D’) the nasal sac. WISH = whole-mount in situ hybridization, KV = Kupffer’s vesicle, hpf = hours post-fertilization, r = retina, ns = nasal sac. (TIF) [file pone.0110484.s005.tif]

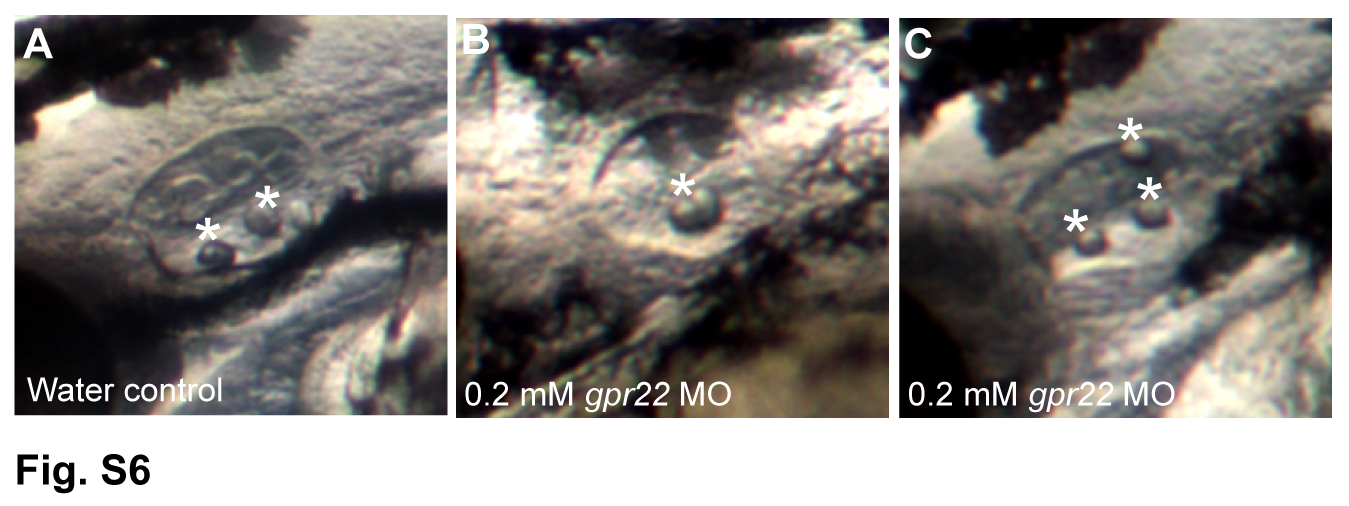

Supplement: Figure S6 — gpr22 morphants display otolith defects. Left ear with anterior to the left of zebrafish embryos at 2 dpf, injected at one cell stage with (A) water or (B, C) 0.2 mM gpr22 MO. (A) Control embryos have 2 tethered otoliths (white asterisks) at the anterior and posterior poles of the otic vesicle. In contrast, gpr22 morphants display (B) fused otoliths or (C) an increased number of otoliths, which are not correctly positioned. dpf = days post-fertilization, MO = morpholino. (TIF) [file pone.0110484.s006.tif]
